# Soil–vegetation moisture capacitor maintains dry season vegetation productivity over India

**DOI:** 10.1038/s41598-022-27277-6

**Published:** 2023-01-17

**Authors:** Dawn E. Sebastian, Raghu Murtugudde, Subimal Ghosh

**Affiliations:** 1 Department of Civil Engineering, Indian Institute of Technology Bombay, Powai, Mumbai 400076 India; 2 Centre for Water Resources Development and Management, Kozhikode, 673 571 Kerala India; 3 Earth System Science Interdisciplinary Center (ESSIC)/DOAS, University of Maryland, College Park, MD USA; 4 Interdisciplinary Program in Climate Studies, Indian Institute of Technology Bombay, Powai, Mumbai 400076 India

**Keywords:** Hydrology, Climate sciences, Ecology, Hydrology

## Abstract

India receives more than 70% of its annual rainfall in the summer monsoon from June to September. The rainfall is scanty and scattered for the rest of the year. Combining satellite data and model simulations, we show that the soil-vegetation continuum works as a natural capacitor of water, storing the monsoon pulse and releasing the moisture to the atmosphere through evapotranspiration over approximately 135 days when the moisture supply from precipitation is less than the evapotranspiration losses. The total Gross Primary Productivity of vegetation in India during the capacitor period accounts for almost 35% of the total annual GPP value. It primarily depends on the soil moisture at the beginning of the period, a measure of moisture capacitance of soil, with a correlation of 0.6. Given that India is the second largest contributor to recent global greening, its soil-vegetation water capacitance plays a significant role in the global carbon balance.

## Introduction

The feedback from land to the atmosphere is driven by Evapotranspiration (ET) which connects the water, energy, and carbon cycles. Approximately 64% of global ET is contributed by transpiration from vegetation^1^. Hence, the transpiration from vegetation plays a vital role in the atmospheric component of the hydrological cycle. The biophysical processes in vegetation that alter the transpiration also control the land–atmosphere carbon dioxide exchanges^2,3^. Hence, the ramification of biophysical changes in vegetation can substantially impact the global and regional climate^4^. Studies show a strong influence of climate variables such as precipitation, temperature, total land water storage, and radiation on vegetation growth and productivity^5,6^. At the same time, the old and diverse forests, irrespective of the vegetation type, play a significant role in dampening the impacts of climate variability on the carbon and hydrological cycles^7^.

Vegetation has a very strong feedback to the atmospheric^8–11^ and hydrological^12^ processes and will play a significant role in the future trajectory of the earth system^13^. Changes in vegetation patterns influence the water yield, especially the low flows, as these can alter the infiltration rates and subsequently soil moisture and groundwater storage^14^. Groundwater can improve the multi-year persistence of rainfall by sustaining evapotranspiration for an extended period of time^15^. Plants may enhance water availability in the future due to decreased transpiration resulting from relatively early stomatal closure at higher CO_2_ concentrations and an increase in soil moisture^16–18^. However, recent studies also show that longer growing seasons with increasing leaf areas due to CO_2_ fertilization and higher evaporative demand of the atmosphere due to warming may increase evapotranspiration^11,12,19–21^. While root zone soil moisture can act as a limiting factor for evapotranspiration^22^, deep-rooted vegetation can uptake water from deeper soil layers to compensate for water deficiencies in top soil layers, thus sustaining evapotranspiration^23^. In the North American Monsoon region, the soil moisture control on evapotranspiration evolves with changes in vegetation and its phenology^24^. The role of the soil-vegetation continuum in driving evapotranspiration is very significant in the context of the inadequacy of Earth System Models (ESMs) in capturing the coupling between soil moisture and evapotranspiration^25–28^. The current ESMs try to capture the various processes that control the land–atmosphere interactions^29^ with the help of observation-aided sophisticated coupling mechanisms; however they still fail to reach a consensus^30–32^. The large spread across the ESMs in modelling these interactions arise due to the complex interactions and feedbacks among the different components of the earth system^33,34^. This highlights the need to accurately monitor these couplings between the different elements; especially a critical process such as ET.

Indian summer monsoon rainfall (ISMR) spans four months from June to September, and contributes to almost 80% of the total annual rainfall over the country while also displaying a very high spatial and temporal variability^35,36^. As can be expected, rainfall impacts the vegetation growth and distribution in the region^37^. With a total geographical area of more than 3.2 million km^2^, India has an extensive vegetation cover: almost 21% forested area and 59% agricultural land^38^. The other vegetation types are shrubs, grasslands, and wetlands^38^. The Indo-Gangetic Plain and Central India, dominated by croplands, are considered global hotspots of land–atmosphere feedback^39^. A likely decline in evapotranspiration and the associated precipitation recycling due to possible deforestation over India may weaken the ISMR^40^. Vegetation also has significant feedback on the onset and the rainfall quantity during the summer monsoon season over the northeastern parts of India^41,42^.

The terrestrial water cycle is strongly connected to the carbon cycle and plays a major role in controlling plant carbon uptake. Tropical forests can mitigate the effects of global warming, both through carbon uptake and evaporative cooling^43^. Globally, the land vegetation absorbs 30% of the atmospheric CO_2_^44^, and hence, its impact on soil moisture dynamics and the feedback in turn to gross primary productivity are critical processes. Being the second highest contributor to the recent global greening, India plays a significant role in terrestrial carbon uptake^45^. Given the strong biosphere feedbacks in the monsoon region^46^ and the enormous spatial and temporal variability associated with ISMR^36,47^, an analysis of the role of vegetation on the water cycle and its impact on the CO_2_ uptake is an imperative. India has two of the eight global hottest biodiversity hotspots^48^, which are natural forests. In addition, a significant fraction of Indian croplands is also rainfed^49^. With a strong seasonal rainfall pattern, the sustainability of rainfed agriculture and forest system during the post-monsoon and other dry seasons lies in the specific characteristics of the Indian soil moisture–vegetation continuum. Such features are not yet explored in the literature to the best of our knowledge. Such properties can also be expected to have a strong implication for the global carbon cycle. Here, we aim to understand the same by analyzing the observed and simulated hydrologic variables and observed Gross Primary Productivity (GPP) data over India.

We have employed the daily gridded precipitation data developed from observed station data^50^ by the India Meteorological Department (IMD) from 1901 onwards. In addition, we have obtained the soil moisture data from the European Space Agency (ESA). The product combines Soil Moisture and Salinity Mission (SMOS) data for the post-2010 period with Advanced Microwave Scanning Radiometer for EOS (AMSRE) data from 2003 to 2010 using neural networks^51^, thus providing a continous dataset from 2003 onwards. This product was developed to include L band-based SMOS soil moisture data into the ESA CCI SM dataset. SMOS products were later included in the ESA CCI soil moisture from v03.2 which was released in 2017. We have used the data for evapotranspiration and its two components viz., soil evaporation, and transpiration, assessed by the Global Land Evaporation Amsterdam Model (GLEAM), version 3. GLEAM uses global measurements, satellite data products, and reanalysis products to develop the different ET components at 0.25° × 0.25° spatial resolution^52,53^ from 1980 to 2015. We have also used the Variable Infiltration Capacity model (details in Methods) for experimental simulations in the study region to identify the plausible factors driving evapotranspiration variability over India. Daily surface downward shortwave radiation and photosynthetically active radiation values provided by the Clouds and the Earth’s Radiant Energy System (CERES) are employed in the study for the period 2001 to 2015. This product incorporates fluxes from geostationary satellites to account for the regional diurnal flux variations between Terra & Aqua CERES satellite measurements^54,55^. The 8-day Gross Primary Productivity (GPP) product (MOD17A2H) at 500 m spatial resolution from Moderate Resolution Imaging Spectroradiometer (MODIS) aboard the Terra satellite^56^ is then employed to understand the association between the hydrological fluxes and the carbon cycle over the region for the period 2001 to 2015.

## Results

### Vegetation as a moisture capacitor

**Figure 1:**
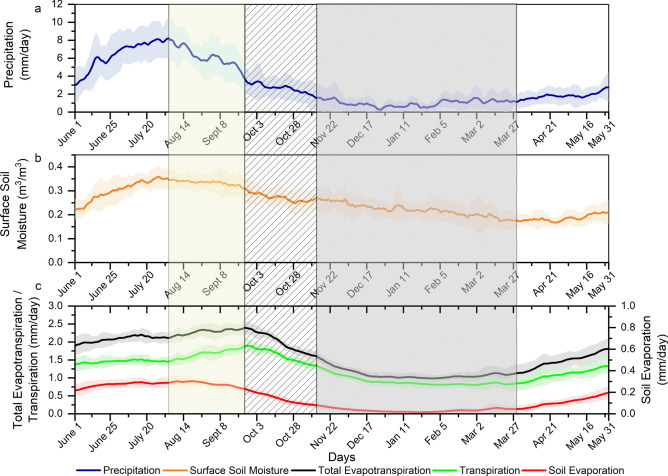
Climatology of observed variables: (**a**) Precipitation, (**b**) Surface Soil Moisture, (**c**) Evapotranspiration and its components for the period 2001 to 2015. The yellow, hatched and grey shaded regions correspond to the delayed response period, pre-capacitor period and capacitor period, respectively. The plots are prepared in Origin 2018.

Figure 1a presents the climatology of observed precipitation averaged over India. It reaches its peak during the first half of August and starts withdrawing in September. Figure 1b shows the climatology of the surface soil moisture, which follows the same pattern as precipitation. This behavior is expected because the satellite soil moisture considers only the top few centimeters of the soil, primarily driven by the precipitation. Figure 1c presents the climatology of ET, and its components averaged over India and it shows that ET continues to increase during the monsoon withdrawal phase. However, soil evaporation immediately starts dropping after the peak of the monsoon and follows climatology of precipitation and surface soil moisture. Transpiration from vegetation drives the increased ET during retreat of the monsoon (Fig. 1c). It is noteworthy that the high ET during the withdrawal of the monsoon is a major contributor to atmospheric moisture loading^47,57^ over the Indian sub-continent. Hence, the role of vegetation in maintaining water cycle during the monsoon withdrawal is surprisingly large. Here, we also aim to understand vegetation’s role in sustaining the land- atmosphere interactions during drier non rainy periods. The ability of vegetation to store water during periods of high precipitation and releasing that water to the atmosphere during drier periods is called moisture capacitance^58^. We define three periods after the peak of monsoon precipitation based on climatology of precipitation and ET. Here, we considered climatology to avoid the low frequency variability associated with the different components of the water cycle within a year. The first period (yellow shaded) between the peaks of precipitation and total ET is termed as the delayed response period. During this period, the increase in total ET and transpiration occurs due to the high moisture availability, moisture supply from precipitation and radiation. The delay here refers to the time-lagged peak of ET with respect to that of the precipitation. The average daily ET experiences an increment of almost 13% during this period despite a 55% decline in precipitation from its peak value. However, this period has a moisture surplus because the precipitation in this period is higher than ET. Studies have shown that the seasonal shifts in vegetation activities and feedback processes over the Indian region are primarily controlled by radiation and precipitation^59,60^. Therefore the increase in ET in this period can be attributed to the high radiation and highlights the role of radiation in driving plant processes during a moisture surplus condition. The precipitation during this period accounts for almost 28% of the total annual precipitation, whereas the ET is approximately 20% of the total annual ET. The daily average incoming radiation is 97% of its annual daily average, meeting the plant demands. The delayed response period lasts for approximately 50 days when spatially averaged over the Indian landmass. The second period (hatched in figures) is the time interval during which ET drops from its peak and precipitation still exceeds ET. We define it as the pre-capacitor period. The pre-capacitor period, which extends for approximately 50 days (based on the climatology of spatial means), witnesses a drop in both precipitation rate (57%) and radiation (18%), leading to a decrease in ET (33%), from the start of the pre-capacitor period. However, the decline in ET is slower than that of precipitation. The total ET during the pre-capacitor period accounts for approximately 17% of total annual ET against a precipitation input of 12% of total annual precipitation. The third period, i.e., the capacitor period (grey shaded in figures), is the phase during which the ET is higher than precipitation. While both precipitation and ET rates decline by approximately 29% when spatially averaged across the Indian landmass, ET during the capacitor period accounts for almost 25% of the total annual ET, which is primarily driven by transpiration (27% of total annual transpiration). Precipitation during the capacitor period accounts for only 12% of total annual precipitation. Intuitively, ET and the associated plant processes cannot be expected to be sustained in a water-limiting condition due to the low precipitation in this period. While factors like surface temperature, radiation and wind speed play a major role in determining ET, the current study does not consider them explicitly. However, the definition of capacitor period implicitly considers the causal factors; for example, the radiation starts increasing after monsoon withdrawal, which is the typical beginning of the capacitor period. The wind also changes its direction with the transition of the season. Hence, for a seasonal climate, the causal factors are built into the definition of the capacitor period. We hypothesis that the ET processes during this period are sustained by the soil-vegetation continuum. The soil and vegetation receive moisture during the monsoon, the delayed response period, and the pre-capacitor period. The stored moisture is released to the atmosphere during the relatively dry capacitor period and supports plant activities. Hence, the soil and vegetation can act as capacitors in the Indian monsoon water cycle. We find that the capacitor days (shaded in grey in Fig. 1) typically extend for ~ 135 days when spatially averaged over India (Fig. 1c).

**Figure 2:**
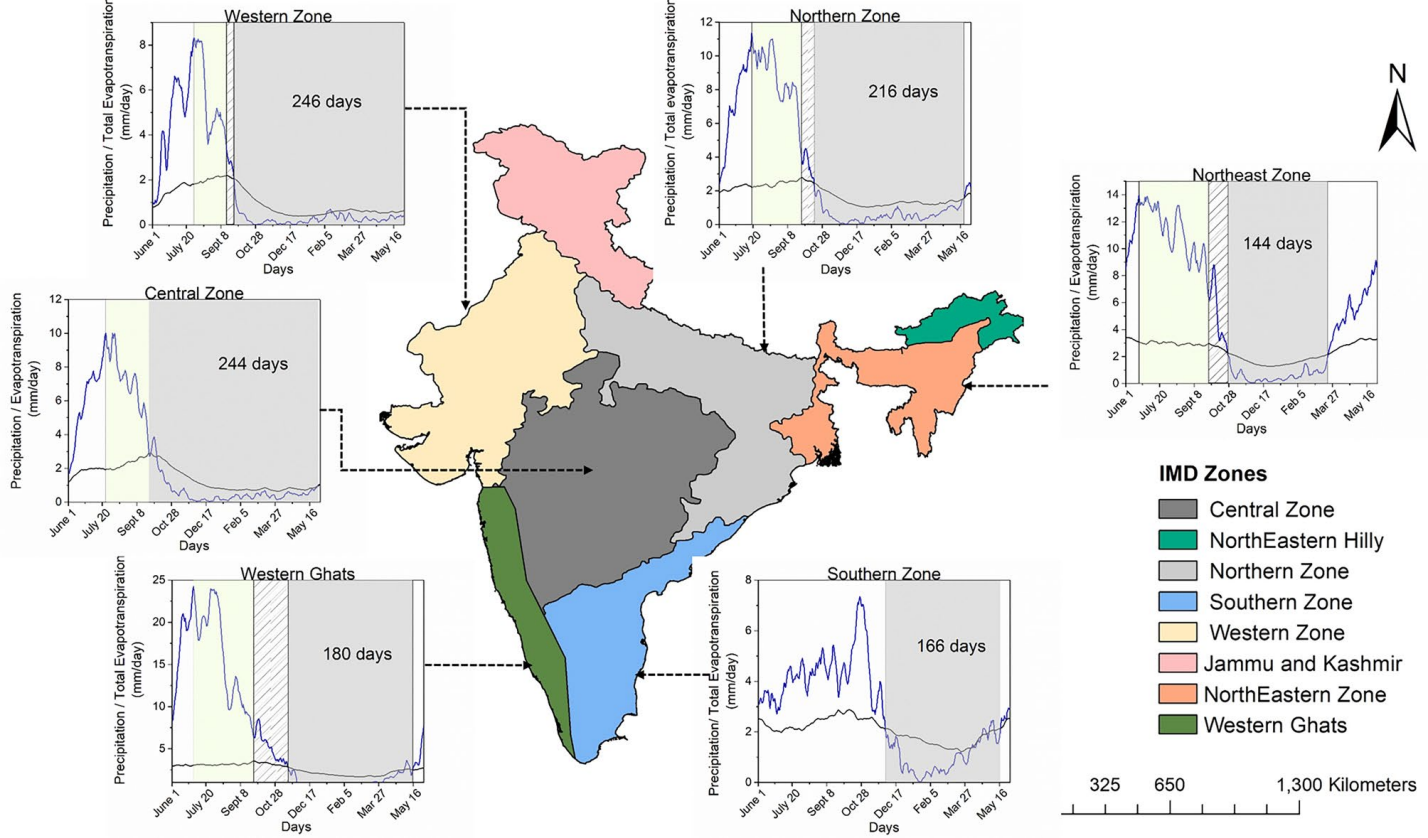
Spatial variability of capacitor days across different homogeneous meteorological subdivisions over India (climatology for the period 2001 to 2015). The shaded yellow, hatched and grey regions correspond to the delayed response period, pre-capacitor period and capacitor period, respectively. The plots are prepared in Origin 2018.

ISMR has a very high spatial variability with approximately 300 mm rainfall over the northwestern desert and 3000 mm in northeast India and the Western Ghats. There is a correspondingly high ecological diversity driven by the climate. Hence, it is imperative to study the spatial variations in the capacitor days. Figure 2 presents the same for different homogeneous meteorological subdivisions over India. The role of high spatial variability of precipitation and ET across the different zones is very clear from the day of the start of capacitor period in the different zones when ET exceeds precipitation. We find that the capacitor days typically vary between 145 and 245 days. We have excluded two regions for our analysis, namely, Jammu and Kashmir and northeast hilly regions. These two regions do not have a well distributed rain-gauge network. Hence, the gridded product may have quality issues. North India, Central India, and the Western zone have capacitor days of more than 200. North India and Central India are the global hotspots for land–atmosphere feedbacks^39^. Hence, the moisture capacitor effect of soil-vegetation may have significant implications on the water cycle over these regions. It has to be noted that the Central zone lacks a pre-capacitor period because of the sudden withdrawal in ISMR after September, reducing the precipitation in the region and a high post-monsoon evapotranspiration rate. The Southern zone of India receives majority of its precipitation during the northeast monsoon season and its impact on the land–atmosphere interactions is visible from the delayed onset of capacitor period. However, since the northeast monsoon season confounds the ISMR withdrawal phase, a period of delayed response or pre-capacitor period could not be defined for the Southern zone. The moisture rich Northeast zone and Western Ghats zone, which receive multiple spells of rainfall across the year have shorter capacitor period. However, both being rich in biodiversity^61^ and Western Ghats being one of the eight global hottest biodiversity hotspots^48^, the role of soil-vegetation continuum in sustaining the land–atmosphere interactions during the dry months is crucial. It has also been reported in Western Ghats region that the vegetation plays a crucial role at the intra-seasonal timescales^58^. We find such capacitor effects continue even during the dry periods and sustain the land atmosphere interactions.

### Role of soil moisture

**Figure 3:**
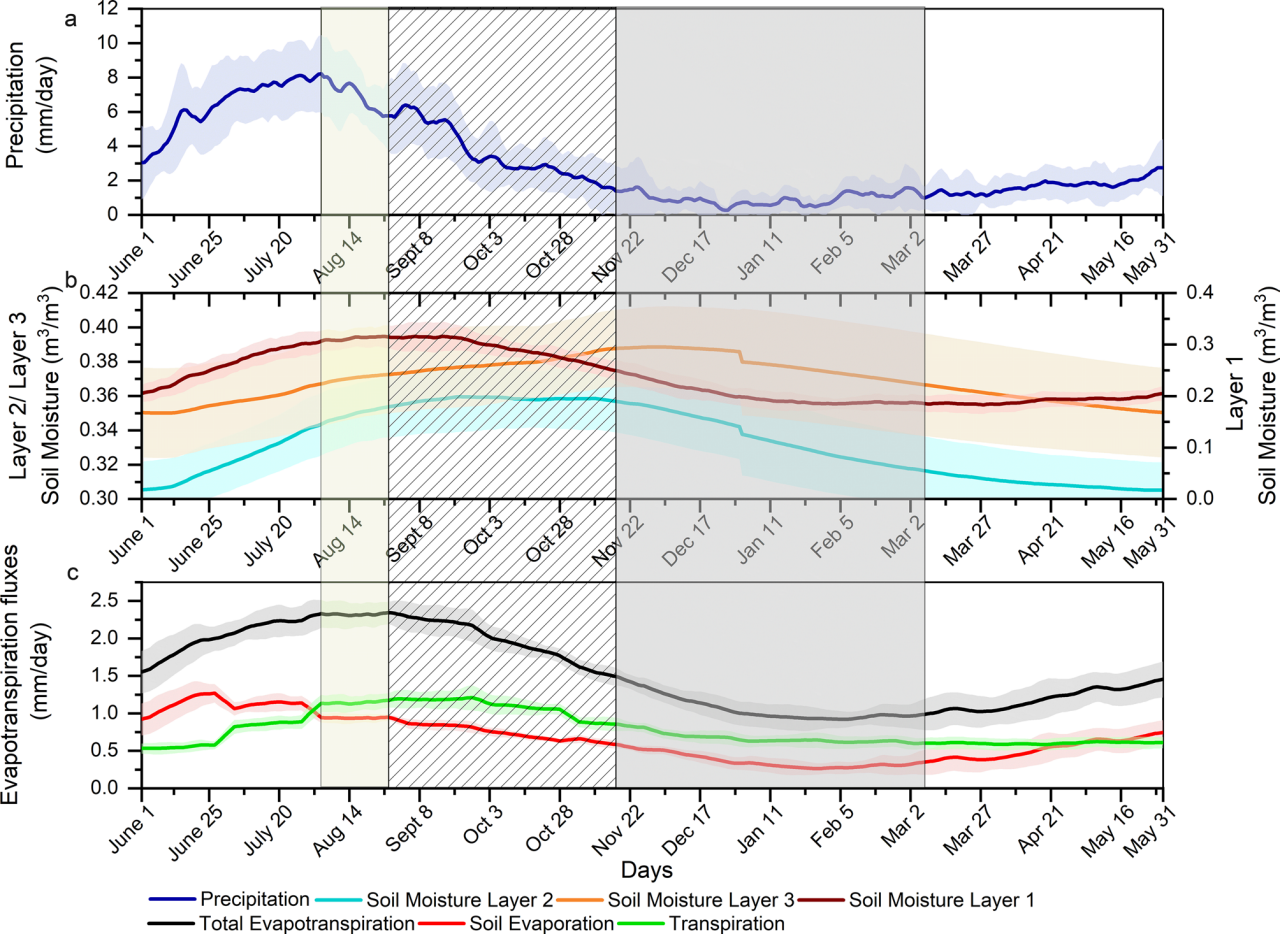
Climatology of (**a**) Observed precipitation (**b**) VIC simulated moisture content in different soil layers, (**c**) VIC simulated total evapotranspiration and its components for the period 2001 to 2015. The shaded yellow, hatched and grey regions correspond to the delayed response period, pre-capacitor period and capacitor period, respectively. The plots are prepared in Origin 2018.

Satellite-derived soil moisture data does not provide any information about the root zone soil moisture. Hence, we cannot use such data to understand the role of soil moisture in the soil-vegetation capacitance. Here, we have performed a hydrological simulation with the Variable Infiltration Capacity (VIC) model for estimating the root zone soil moisture (details in methods). The model considers 3 layers of soil. The top layer extends from the surface to 0.3 m depth. The thickness of the middle and the bottom layers are approximately in the range of 1 m–2.5 m and 0.2 m, respectively. For majority of the crops, the root zone lies in the 2nd layer of soil. The 3rd layer represents a deeper soil layer. VIC can simulate ET and surface soil moisture satisfactorily, averaged over India as seen in Supplementary Fig. 1. The top layer soil moisture peak is slightly delayed in the simulation compared to the satellite data which is expected because the simulated soil moisture is for the 30 cm deep top layer, and the satellite estimates are for less than 5 cm. The simulated climatology of the components of ET and soil moisture for different layers are presented in Fig. 3. The simulated patterns also track observations closely, with transpiration contributing to the delayed peak in ET. The soil evaporation drops somewhat early in the simulations. We use the same approach as in Fig. 1 to identify the capacitor days. In the simulation, we find the number of capacitor days is ~ 110, agreeing with the observations. We also find that the simulated soil moisture of layer 2 starts declining faster during the capacitor period. As layer 2 represents the root zone, we conclude that the soil moisture actively participates in maintaining ET and other vegetation processes, thus decaying faster. The root zone soil moisture that is being fed by precipitation during the monsoon, the delayed response period and the pre-capacitor period are then utilized by the vegetation to maintain land–atmosphere interactions. We further observed a correlation of 0.63 between the total evapotranspiration loss during the capacitor period and the available soil moisture (sum of layer 1 and 2) at the beginning of the capacitor period. Soil moisture in the deeper layer varies much less with limited seasonal variations. The regional plots similar to Fig. 3 are presented in Supplementary Figs. 2–7.

VIC simulates almost similar spatial variations across all the regions with capacitor period varying from 133 to 246 days. VIC underestimates the number of capacitor days for Western Ghats, which might be attributed to the undulating terrain of the region not adequately represented in the model. However, the regional contrasts are clearly represented and the role of root zone soil moisture is distinctly visible in all the regions. Larger depletions of root zone soil moisture were observed in the Northern and Central zones, highlighting the dependence of vegetation in these areas on the root water uptake. These two zones being global hotspots of land–atmosphere feedbacks further amplifies its relevance. The Western Ghats and Northeast zones, which receive precipitation during pre-monsoon season experience shorter capacitor periods, and thus less depletion of root zone soil moisture. The Western zone with large areas under desert or scarce vegetation also show a lower rate of depletion of root zone soil moisture.

### Gross primary productivity during the capacitor days

**Figure 4:**
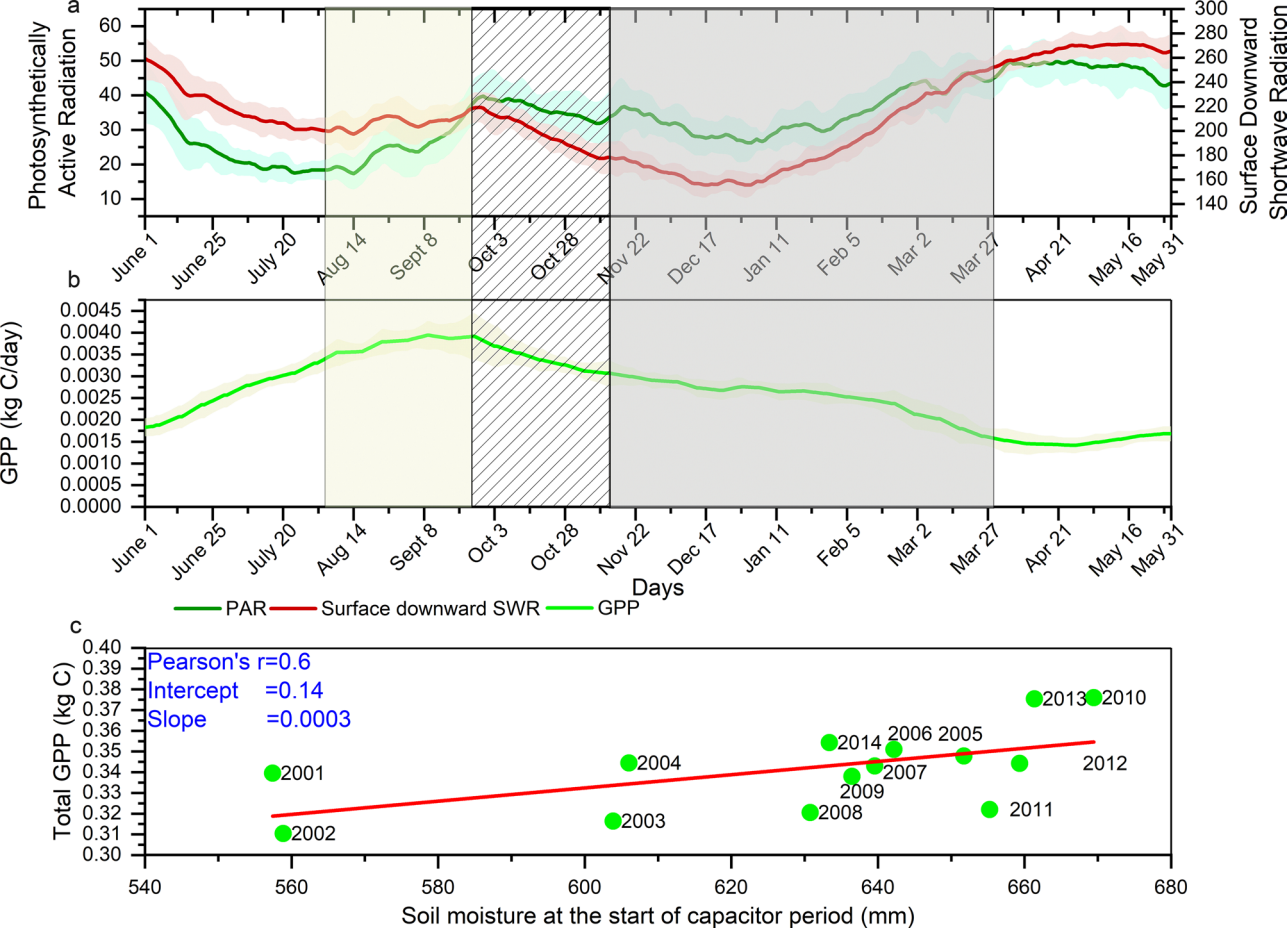
(**a**) Climatology of Photosynthetically Active Radiation (PAR) and downward surface Shortwave Radiation (surface SWR) for 2001 to 2015. (**b**) Climatology of GPP for 2001 to 2015. The shaded yellow, hatched and grey regions correspond to the delayed response period, pre-capacitor period and capacitor period, respectively. (**c**) Association between soil moisture at the beginning of the capacitor period and total GPP generated during the period. A linear correlation of 0.57 (statistically significant at p = 0.05) is observed between the two variables. The plots are prepared in Origin 2018.

After the peak of the monsoon, the retreat and subsequent withdrawal of the monsoon result in clear sky conditions with increased surface radiation. During the delayed response period, high radiation with enhanced water storage in the soil–plant continuum, results in greater GPP by 15% from its value at the start of the period with faster absorption of atmospheric CO_2_^62^ (Fig. 4b). We found a similar result using the variable Photosynthetically Active Radiation (PAR, Fig. 4a). During the pre-capacitor and capacitor periods, the GPP declines due to a reduction in precipitation. This is expected because precipitation is the most dominant climate driver of GPP^63^. However, vegetation continues to uptake carbon even during the precipitation-deficit capacitor period, resulting in a GPP growth during this period, accounting for approximately 35% of the total annual GPP. Notably, the precipitation during this period accounts for only 12% of its yearly total. Upon further analysis (Fig. 4c), we observed that the GPP during the capacitor period exhibits a strong correlation of approximately 0.6 with the soil moisture at the beginning of this period. We considered the sum of the top and middle layer soil moisture at the beginning of capacitor period for this correlation estimation. We infer that the GPP is sustained during this period by the stored soil moisture generated from rainfall during monsoon, delayed response and pre-capacitor periods. By highlighting the role of soil moisture-vegetation continuum on land–atmosphere interactions, our study emphasizes its capacity to act as a buffer to store water on land even after the decline in monsoon precipitation and then use it during dry periods. Global studies also suggest the role of soil moisture in controlling the carbon uptake variability^3^; however, such analyses were not performed in the present context. Precipitation during the capacitor days also has a strong correlation of 0.7 with GPP. However, the total precipitation is not sufficient to supply the plant water requirement (Fig. 1), since ET (the water loss from soil) is higher than precipitation (water supply to soil). This water balance further highlights the role of soil moisture in sustaining the GPP.

Regional analysis of GPP across different homogeneous meteorological subdivisions with their varied vegetation cover are consistent and similar to those observed for Indian landmass (Supplementary Fig. 8). However, the GPP climatology varies across the subdivisions depending on its meteorological and vegetation characteristics. The Northeast and Western Ghats zones are rich in vegetation with extensive forest covers. The GPP over these regions is sustained quite well in the capacitor period, with a slight drop followed by recovery. However, the Northern, Central and Western regions experience a very low GPP by the end of the capacitor period, although periods of stable/increasing GPP are visible in Northern and Western zones which might be attributed to the influence of westerly disturbances^64^ and agriculture respectively, in the two regions. It is also interesting to note that the peak GPP in the Southern zone almost coincides with the inception of the capacitor period, which indicates a strong dependence of GPP on precipitation in the region. The other reason behind the spatial variations of GPP patterns across subdivisions is the variations in the surface downward shortwave radiation, as depicted in Supplementary Fig. 9. This finding further demonstrates the synergistic contribution of radiation and moisture during the capacitance period in driving plant productivity, with soil moisture at the inception of the capacitor period driving it across the Indian landmass. Zonal level correlation analysis of the capacitor period GPP and the initial soil moisture further reinforces this argument (Supplementary Fig. 10). The role of the initial soil moisture on the capacitor period GPP is prominent across the subdivisions with statistically significant correlations.

## Discussions

The Indian landmass is known to be one of the global land–atmosphere hotspots^39,57^, further augmented by high irrigation. Though the land–atmosphere feedback studies highlight the role of ET in sustaining the retreating monsoon, there is a lack of analysis on understanding the processes sustaining the ET and plant productivity after the monsoon and the major cropping season. We explored the same in the present work, which is unique and unreported in the literature to the best of our knowledge. We found that the water stored by the soil–plant continuum drives the post-monsoon terrestrial water cycle over India. This stored water sustains the plant's requirements for the rest of the year during periods of reduced precipitation. The capacitance of soil–plant continuum maintains ET, productivity, and land–atmosphere feedback by exploiting this water storage capacity.

Our results not only explain the post-monsoon vegetation productivity in India, but also can provide the foundation for understanding the dry season plant productivity in other monsoon regions such as Australia, South America and Africa. Implications of these processes for global land–atmosphere feedbacks, carbon cycle and vegetation responses in a warming world can hardly be overemphasized. The tropical monsoon regions absorb a significant proportion of atmospheric CO_2_. Earlier literature^2,3^ have indicated the role of soil moisture and total water storage in controlling the variability of plant carbon uptake. Our work further strengthens the hypothesis on the role of water storage on plant productivity. The present work will be further extended to the global monsoon regions to elicit the soil–plant continuum moisture capacitance and the associated storage for water and the role they play in the terrestrial carbon uptake.

One of the major caveats of the current study arises from the lack of continuous hydrological observations for soil moisture and evapotranspiration in the region. The satellite-based soil moisture represents only the top few centimetres of the soil, which might not be able to provide the exchange of water between the layers and into the atmosphere through vegetation. Furthermore, the GLEAM algorithm considers only the top layer soil moisture for computation of ET fluxes, thus neglecting the role of both irrigation and vegetation interaction with the atmosphere. Such processes cannot be neglected for the study region with vast areas under irrigation. Moreover, the validations of AMSRE-SMOS soil moisture product and the GLEAM products are insufficient due to the lack of observation stations in India. The other major limitation of the study arises from the MODIS GPP product, which does not account for the carbon fertilization. Hence, extensive spatially distributed observation stations and flux towers are required to accurately capture the hydrological and vegetation variables for a long period. The VIC model employed in the current study also does not take into account the impact of irrigation or vegetation variability, which are crucial for the study region with extensive vegetation heterogeneity and an irrigation-dependent agricultural system^65^. Hence, future works need to be directed at understanding the impact of irrigation and variable vegetation on the capacitor period of a region along with the regional variability. The soil moisture—vegetation capacitor effect is a robust finding which highlights the data needs to further quantify its impact on the physical climate and the carbon cycle.

## Conclusion

By highlighting the role of soil moisture-vegetation continuum on land–atmosphere interactions, our study emphasizes its capacity to act as a buffer to store water on land even after the decline in monsoon precipitation and then use it during dry periods. Hence, the role of vegetation in sustaining the moisture supply to the atmosphere is pivotal. Capacitor property of vegetation relies on the region's climate and vegetation types and distributions, as evident from its variability between 145 to 245 days across the different climate zones in India. We have shown that the capacitance effect of vegetation likely drives stronger land atmosphere feedbacks in some of these regions. Modelling studies further support the capacitance property of Indian vegetation, which is reinforced by the moisture content in the root zone.

Given that India is the 2nd highest contributor to the recent global greening, we find that the role of the capacitor days in understanding the land’s carbon sequestration potential is enormous, especially as an additional contributor to the Nationally Determined Contributions as well as for enhancing soil health and crop yields. Our results also call for the analysis of vegetation moisture capacitance properties over the other monsoon regions, such as the Amazon forest in South America. Finally, the strong association between the water and the carbon cycle over India calls for stronger interactions between the hydrological and biogeochemical scientific communities towards developing regional land surface models for South Asia.

## Methods

The gridded precipitation data from IMD, combined AMSRE-SMOS soil moisture data and ET fluxes from GLEAM were first extracted and spatially averaged to determine the climatology for the Indian landmass. To understand the spatial variability in the ET response, we have considered spatial average over the meteorologically homogeneous regions in India^66^. The Variable Infiltration Capacity (VIC) model have been employed for modelling the interaction of deeper soil layers on ET. VIC is a semi-distributed mesoscale hydrological model. It integrates water and energy balance equations on discrete grids^67^. VIC can compute land and atmospheric fluxes based on water and energy balance at daily/sub-daily time steps. In the current study, VIC is run at a daily time scale with a grid size of 0.5° × 0.5°. Meteorological inputs for precipitation, maximum and minimum temperature are obtained from IMD. Wind velocity is obtained from ERA Interim reanalysis. The different meteorological inputs are then converted to the model grid resolution. Grid-wise meteorological input files including daily time series of the three input variables, are provided as input for the VIC simulation. Each grid cell in VIC is divided into smaller tiles, covered by different land cover types to account for the sub-grid heterogeneity. The variation of vegetation types across each grid and their root distribution is represented in the vegetation parameter file developed from the Land Use Land Cover (LULC) Map provided by MODIS (MCD12Q1)^68^ following the International Geosphere-Biosphere Programme (IGBP) classification. Vegetation parameters for the different vegetation types used in VIC are provided in the vegetation library file. Here, we obtained the climatology of vegetation property using the values of Leaf Area Index (LAI) obtained from MODIS Terra satellite (MOD15A2H)^69^. We have used the albedo from MODIS product MCD43A3^70^ and fraction of vegetation cover derived from the MODIS Normalized Difference Vegetation Index (NDVI) product MOD13Q1^71^ based on a linear relationship^72^. Soil properties are considered to be the same throughout each grid cell. Soil parameter file, specifying the soil properties is developed from the global soil parameter file provided with the VIC model based on the soil map from the Food and Agricultural Organization. VIC is suitable for studying the variation in soil moisture capacity at sub-grid level, the nonlinear recession of base-flow, and topography. In the current study, we consider 3 soil layers. While variable infiltration capacity parameters control infiltration into the top soil, moisture loss from top layers to the atmosphere is mainly aided by soil evaporation. The potential evapotranspiration is calculated using Penman–Monteith equation considering it as a function of vapour pressure deficit and net radiation. The interception of rainfall by the canopy is calculated as a function of LAI. Runoff computation is based on the bottom layer. One of the major drawbacks of VIC model is that it does not consider inter-grid non-channel flow and water can enter into a grid cell only from the atmosphere. All the figures in the manuscript have been plotted using Origin 2018 after applying a seven day moving window average. The Pearson’s correlation coefficient between total GPP and soil moisture at the beginning of the capacitor period is determined in MATLab 2020.

## Supplementary Information

Supplementary Information 1.

Supplementary Information 2.

## Data Availability

Gridded precipitation and temperature data provided by IMD is available at IMD website (https://www.imdpune.gov.in/Clim_Pred_LRF_New/Grided_Data_Download.html). Evapotranspiration products from GLEAM model are available at model website (https://www.gleam.eu/). SMOS level 4 land research product combining SMOS and AMSRE soil moisture can be downloaded from Centre Aval de Traitement des Données SMOS (CATDS) website, which is the French ground segment for SMOS level 3 and 4 data (https://www.catds.fr/Products/Available-products-from-CEC-SM/L4-Land-research-products). The various MODIS satellite land products can be obtained from Land Processes Distributed Active Archive Centre (LP DAAC) (https://lpdaac.usgs.gov/). The VIC simulations will be made available. CERES radiation data can be downloaded from CERES website (https://ceres.larc.nasa.gov/data/).
