# Supplementary material for: Soil–vegetation moisture capacitor maintains dry season vegetation productivity over India
Source: Sci Rep. 2023 Jan 17;13:888. doi: 10.1038/s41598-022-27277-6 (PMC9845320; doi:10.1038/s41598-022-27277-6)
Supplement: Supplementary file 1 — Supplementary Information 1. [file 41598_2022_27277_MOESM1_ESM.docx]

**Supplementary Information**

**for**

**Soil - Vegetation Moisture Capacitor Maintains Dry Season Vegetation Productivity over India**

Dawn E. Sebastian,^1,2^ Raghu Murtugudde,^3,4^ Subimal Ghosh^1,4,*^

^1^Department of Civil Engineering, Indian Institute of Technology Bombay, Powai, Mumbai – 400076, India.

^2^ Centre for Water Resources Development and Management, Kottayam Sub-centre, Kottayam Main P O - 686 001, Kerala, India.

^3^Earth System Science Interdisciplinary Center (ESSIC)/DOAS, University of Maryland, College Park, MD, United States of America.

^4^ Interdisciplinary Program in Climate Studies, Indian Institute of Technology Bombay, Powai, Mumbai – 400076, India.

*Correspondence to Subimal Ghosh, [subimal@civil.iitb.ac.in](about:blank)

This supplementary information includes Supplementary Figures 1 to 9 and Supplementary Table 1.


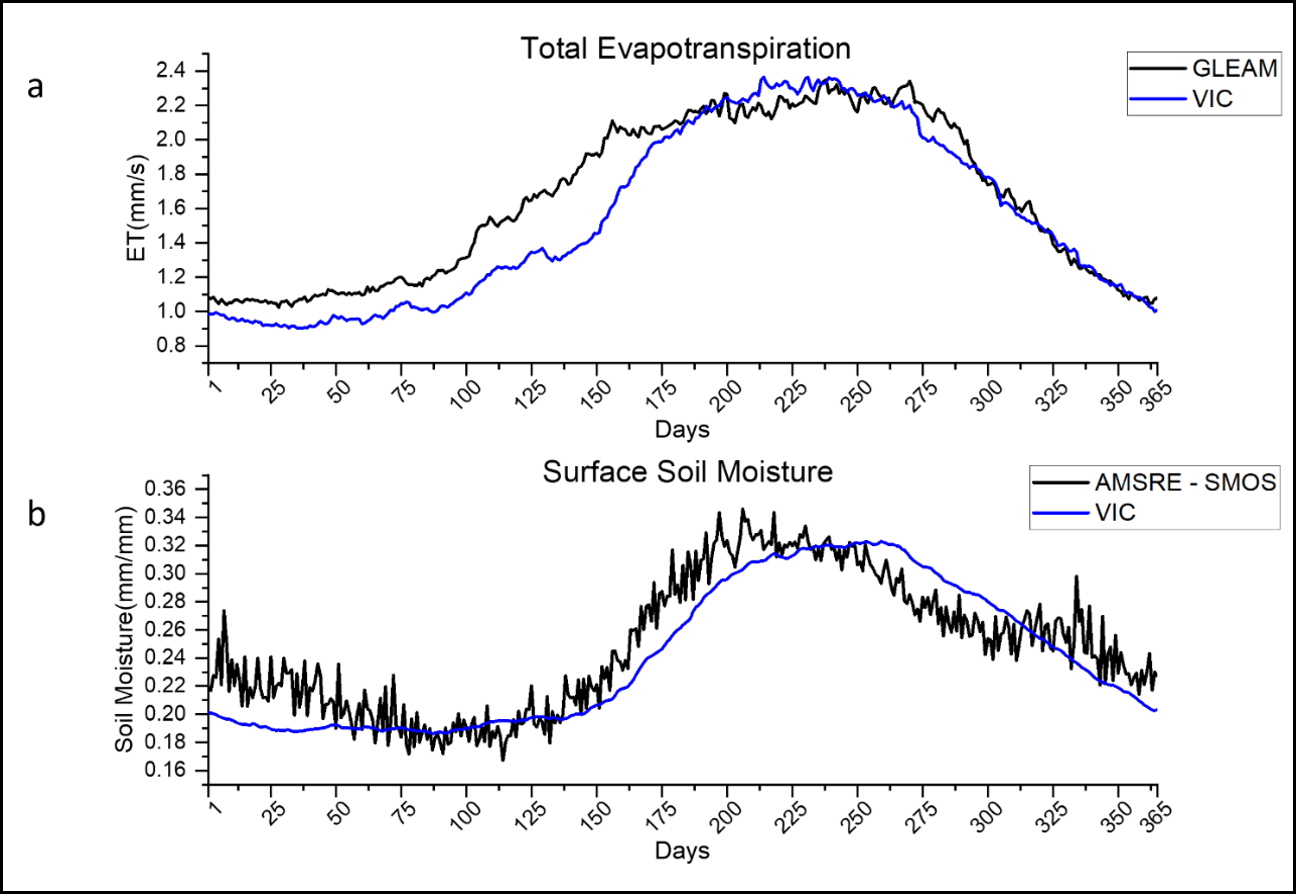


Supplementary Figure 1. Validation of VIC Model Simulations: (a) Observed (GLEAM) and Simulated Total Evapotranspiration, (b) Top layer soil moisture simulated by VIC and the observed surface soil moisture from SMOS-AMSRE combined product. The climatology was computed for the period between 2001 and 2015 and plotted using MATLab 2020.





Supplementary Figure 2. Climatology of (a) Observed precipitation and simulated variables, (b) Moisture Content in different soil layers, (c) Total Evapotranspiration and its components for Central India for the period between 2001 and 2015. Vegetation capacitor days are shaded grey in the figure with delayed response period and pre-capacitor period represented as yellow and hatched, respectively. The plots are prepared using Origin 2018.





Supplementary Figure 3. Climatology of (a) Observed precipitation and simulated variables: (b) Moisture Content in different soil layers, (c) Total Evapotranspiration and its components for Northeast India for the period between 2001 and 2015. Vegetation capacitor days are shaded grey in the figure with delayed response period and pre-capacitor period represented as yellow and hatched, respectively. The plots are prepared using Origin 2018.





Supplementary Figure 4. Climatology of (a) Observed precipitation and simulated variables, (b) Moisture Content in different soil layers, (c) Total Evapotranspiration and its components for Northern India for the period between 2001 and 2015. Vegetation capacitor days are shaded grey in the figure with delayed response period and pre-capacitor period represented as yellow & hatched, respectively. The plots are prepared using Origin 2018.





Supplementary Figure 5. Climatology of (a) Observed precipitation and simulated variables, (b) Moisture Content in different soil layers, (c) Total Evapotranspiration and its components for Western India for the period between 2001 and 2015. Vegetation capacitor days are shaded grey in the figure with delayed response period and pre-capacitor period represented as yellow and hatched, respectively. The plots are prepared using Origin 2018.





Supplementary Figure 6. Climatology of (a) Observed precipitation and simulated variables, (b) Moisture Content in different soil layers, (c) Total Evapotranspiration and its components for the Western Ghats for the period between 2001 and 2015. Vegetation capacitor days are shaded grey in the figure with delayed response period and pre-capacitor period represented as yellow and hatched, respectively. The plots are prepared using Origin 2018.

*

*

Supplementary Figure 7. Climatology of (a) Observed precipitation and simulated variables, (b) Moisture Content in different soil layers, (c) Total Evapotranspiration and its components for the South India for the period between 2001 and 2015. Vegetation capacitor days are shaded grey in the figure with delayed response period and pre-capacitor period represented as yellow and hatched, respectively. The plots are prepared using Origin 2018.





Supplementary Figure 8. Increase in GPP during the capacitor days (shaded in grey) over different meteorological subdivisions in India. Climatology of GPP is computed for the period between 2001 and 2015. The plots are prepared using Origin 2018.





Supplementary Figure 9. Climatology of Photosynthetically Active Radiation and Surface Downward Shortwave Radiation over different meteorological subdivisions in India for the period between 2001 and 2015. The plots are prepared using Origin 2018.





Supplementary Figure 10. Association between soil moisture at the beginning of the capacitor period and total GPP captured during the period for different meteorological subdivisions in India for the period between 2001 and 2014. The plots are prepared using Origin 2018.

Supplementary Table 1 Pearson’s correlation of Soil Moisture at the Start of the Capacitor Period and Total GPP Captured during the Period for Different Regions

| Sl. No | Region | Pearson’s r | Significance Level (%) |
| --- | --- | --- | --- |
| 1 | India | 0.57 | 95 |
| 2 | Central Zone | 0.66 | 99 |
| 3 | Northeast Zone | 0.56 | 95 |
| 4 | Northern Zone | 0.52 | 95 |
| 5 | Southern Zone | 0.64 | 99 |
| 6 | Western Zone | 0.82 | 99 |
| 7 | Western Ghats | 0.75 | 99 |
